# Prevalence of cardiac implantable electronic device infections in Germany in 2015

**DOI:** 10.1038/s41598-024-82622-1

**Published:** 2024-12-16

**Authors:** Benito Baldauf, Reinhard Vonthein, Ernest W. Lau, Marzia Giaccardi, Ojan Assadian, Philippe Chévalier, Christelle Haddad, Kerstin Bode, Andreas Klöss, Roberto Cemin, Hendrik Bonnemeier

**Affiliations:** 1 Institute of Life Sciences, Hochschule Bremerhaven, An der Karlstadt 8, 27568 Bremerhaven, Germany; 2 Medical Faculty, Christian-Albrechts University, Christian-Albrechts-Platz 4, 24118 Kiel, Germany; 3 Institut für Medizinische Biometrie und Statistik, Universität zu Lübeck, Ratzeburger Allee 160, 23562 Lübeck, Germany; 4 Department of Cardiology, Royal Victoria Hospital, Grosvenor Road, Belfast, BT12 6BA UK; 5 Department of Cardiology, Ospedale Santa Maria Annunziata, Ponte a Niccheri, 50012 Florence, Italy; 6 Regional Hospital Wiener Neustadt, Corvinusring 3-5, 2700 Wiener Neustadt, Austria; 7 Institute for Skin Integrity and Infection Prevention, School of Human and Health Sciences, University of Huddersfield, Huddersfield, HD1 3DH UK; 8 Department of Cardiology, Hôpital Louis Pradel, 59 Bd Pinel, 69500 Bron, France; 9 Department of Electropyhsiology, Herzzentrum Leipzig, Strümpellstraße 39, 04289 Leipzig, Germany; 10 Research Institute of the Local Health Care Funds, Berlin, Germany; 11 Intensive Care Unit, Ospedale Regionale San Maurizio, Via Lorenz Böhler 5, 39100 Bolzano, Italy; 12 University Medical Centre Rostock, Schillingallee 35, 18057 Rostock, Germany

**Keywords:** Epidemiology, Cardiac implantable electronic device infection related procedures, CIED infection mortality rate, Cardiology, Diseases, Health care

## Abstract

The placement of cardiovascular implantable electronic devices (CIED) comes with a risk of adverse events (AE). AE comprise complications associated with the procedure and the device. CIED infection is a severe AE. Reported CIED infection rates are regionally differing and publications of contemporary large cohort data regarding CIED infections for Germany are lacking. This investigation provides rates of CIED infection related hospital admission and CIED infection related mortality in Germany. Data from Germany’s largest healthcare insurance fund was screened for CIED procedures during the period: January to December 2015. These data included placement, revision with aim to up- or downgrade, generator substitution, early revision and partial or complete extraction using specific procedure codes for reimbursement. Among the total population of beneficiaries undergoing CIED procedures, International Classification of Diseases codes (ICD 10 GM), operational and procedural codes for partial or complete hardware removal were used to identify CIED infections and lead related endocarditis. To report the corresponding mortality rates, the hospital discharge status was documented. In 2015, from a population of more than 27 million beneficiaries, we identified the cohort of 60,296 patients (57.3% male) who underwent any invasive CIED procedure. Within three months from the index procedure, 1,595 patients developed a major CIED infection. 1,129 (1.87%) were related to the generator pocket and 466 (0.8%) were related to the transvenous proportions of the leads, mortality after those infections was 8.4% and 15.24%, respectively. In summary, our data reveals a higher prevalence of acute CIED infection than previously noted. The elevated number of additional procedures in cases of CIED infection implies a deficiency in infection awareness.

**Supplementary Information:**

The online version contains supplementary material available at 10.1038/s41598-024-82622-1.

## Introduction

Reported CIED infection rates are inconsistent^1^. Observation intervals, patient characteristics, geographical locations and different definitions are all reasons for these variations. The largest clinical trials and observational studies to date report a CIED infection rate of 0.7 to 4.2%^2–8^.

Currently, the risk of CIED infection in de novo implantation is estimated to be around 0.5-0.7%^9^. Procedure, device and patient-specific factors significantly increase the risk of CIED infection^10^. CIED infections not only come with the risk of increased morbidity, they also increase the risk for mortality. Reported mortality rates are 4–13.7% ^6,11,12^ and may exceed 25%^13,14^ in the first year after the infection.

Procedures involving the revision and extraction of CIEDs due to infections have a significant financial burden on both healthcare systems and patients^15–17^.

As of now, there is a dearth of data documenting the prevalence of CIED-associated infections in Germany. Our extensive observational study was undertaken to provide insight into the incidences of acute CIED infection and the corresponding mortality rates in the country.

The aim of our study is to offer an overview of the acute rates of major CIED infection, with stratification to localized CIED infection, and lead-related endocarditis. Simultaneously, it was designed to document the total rate of CIED procedures and mortality rates after treatment of localized CIED infection and lead-related endocarditis.

## Methods

### Study design

For this observational study, de-identified nationwide administrative claims data was used from the “Allgemeine Ortskrankenkassen (AOK)”, the largest health insurance group within the statutory health insurance system in Germany. Due to the use of strictly anonymized data and the resulting application of EU recital 26, ethical approval was waived.

### Participant data collection

AOK provides statutory health insurance for 24,495,897 beneficiaries representing 34,6% of the German health insurance population totaling 70,728,398 members in 2015^18^. At the same time the AOK provides service for more than 27 million members (including non-paying members such as family). The German population was 82,175,684 in 2015^19^. Membership in the statutory healthcare funds or insurance schemes is available to all German citizens and individuals holding permanent residence status, regardless of professional affiliation, income level, age, or pre-existing comorbidities. However, civil servants, high-income families, and the self-employed are underrepresented, as they may opt to receive services through private healthcare funds in Germany.In Germany, health insurance is obligatory to the majority of its inhabitants. According to the German billing method for the health care system (§ 301 of the German social insurance code volume 5 (§ 301 SGB V)), the service provider (hospital) is legally obliged to report all diagnoses, procedure codes (OPS) and results to the health insurance funds. Therefore, the annual anonymized dataset contains detailed information on patient characteristics (including age, gender, diagnoses, procedure codes, length of hospital stays, individual state at hospital discharge: deceased or alive).

In Germany, medical reimbursement within the public healthcare sector is determined by a base cost value (BBFW), which represents a federal monetary benchmark for calculating the cost per case. This base value is multiplied by the center’s case mix index (CMI) and the Disease Related Groups (DRG) code system multiplier. The federal BBFW is subject to annual adjustments; for the year 2015, it was established at €3,231.20^20^. Given its connection to reimbursement, the data quality is considered high. All submitted data are internally verified for consistency. In cases of inconsistency, the data are reviewed in hospitals by the “Medizinischer Dienst " (MD), the medical consulting and assessment service of statutory health insurance. The MD ensures equal access to healthcare benefits based on objective medical criteria, as mandated by law, thereby ensuring that reimbursement criteria are met^21^.

### Procedures

CIED-related procedures were defined by the following OPS codes: 5-377 and following, 5-378 and following and 5-934 and following for de novo placement of permanent pacemakers (PPM) or implantable cardioverter defibrillators (ICD), cardiac resynchronization therapy (CRT), cardiac contractility modulation (CCM), subcutaneous ICD (S-ICD), generator replacement, revisions for upgrading or downgrading the CIED, and extraction or repositioning of CIED hardware.

**Fig. 1:**
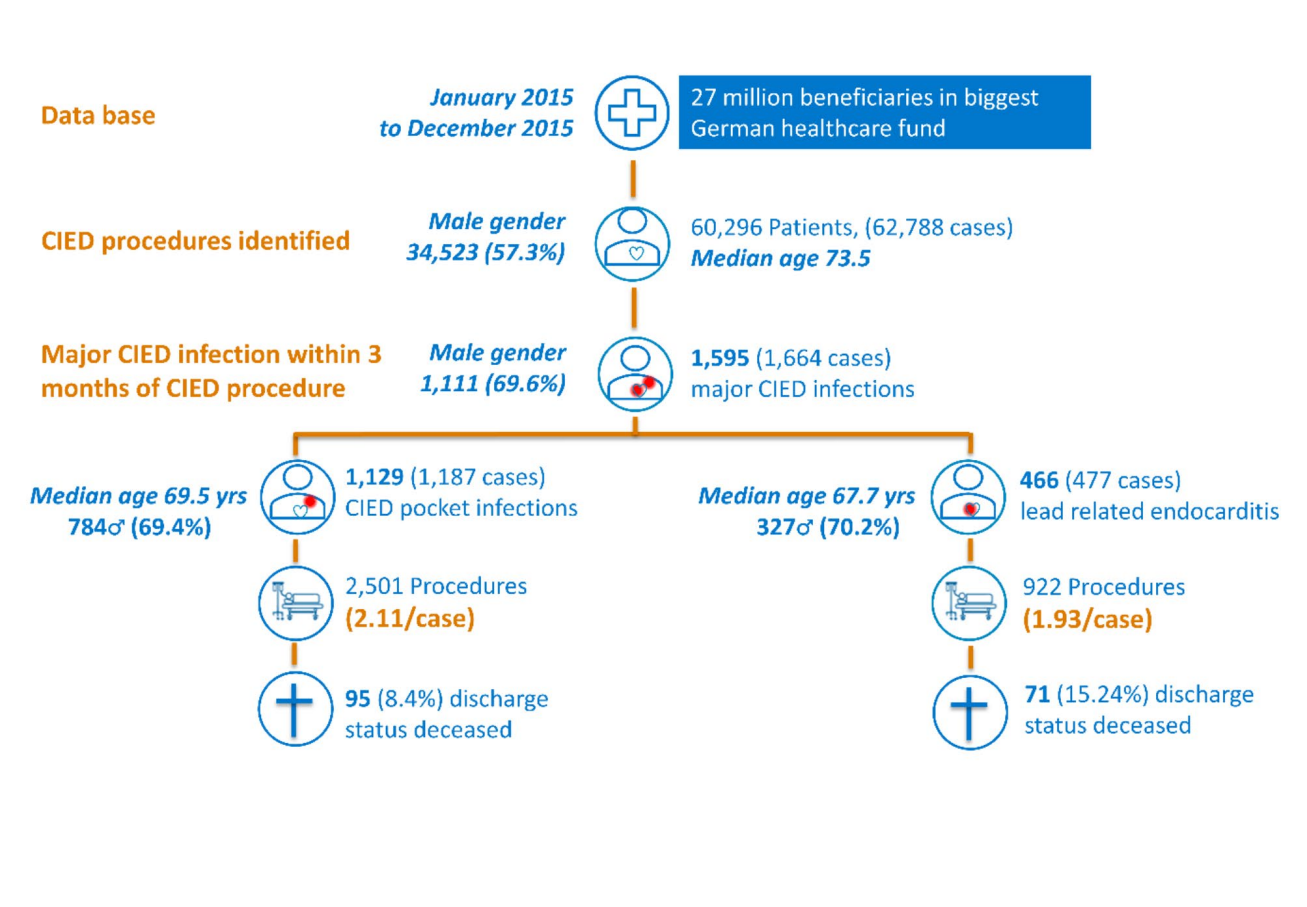
Identification of CIED procedures and CIED related infections and lead-related endocarditis in our cohort.

CIED infections were identified by OPS codes for CIED procedures and international classification of diseases version 10 German modification (ICD-10 GM) code T82.7 for infection. Lead-related endocarditis was identified using ICD-10 GM codes for endocarditis (I33.0, I38, I39) and OPS codes for device removal in patients with previous CIED placement or revision. Mortality data, recorded as discharge status or end of membership, were retrieved for 2015, resulting in a median follow-up of less than six months. The overall mortality rate was not comparable to general mortality due to a hazard peak near the intervention time (Figs. 1, S1; Table S1)

### Statistical analysis

All analyses were done using Oracle Database (Database 12cR1) and statistics software R (version 4.3.0). 95%-confidence intervals for proportions and their differences were calculated by the score method using R package PropCIs 0.3-0 and similarly for relative risk (RR) with package ratesci 0.4-0.

## Results

### Procedures and population

During the study period (January 2015 – December 2015) a total of 78,177 CIED procedures were conducted in 60,296 distinct patients. 34,523 (57.3%, 95%CI 56.9–57.7%) were male. 62,788 cases were reimbursed. Median age for hospital admission was 73.5 years. 746 patients or 1.24% (95%CI 1.15–1.33%) of the total cohort succumbed during 2015, i.e., with median follow-up near 0.5 years. This is near half the national one-year mortality of 2.43% at that age considering the gender proportions. The total number of patients undergoing a CIED procedure, the number of cases reimbursed, the total number of procedures reimbursed, gender-distribution, median age at hospitalization and overall mortality are displayed in Table 1. The overall mortality rate was not comparable to general mortality due to a hazard peak near the intervention time.

**Table 1:** Population, gender, median age and discharge status deceased.

Beneficiaries	Patients	Cases	Procedures (per case)	Male gender (%)	Median age	Deceased (%)
24,495,897 (> 27 Mio incl. family members)	60,296	62,788	78,177 (1.25)	34,523 (57.3)	73.5	746 (1.24)

A major CIED infection (comprising localized CIED pocket infection and lead-related endocarditis) within three months from the index procedure was diagnosed in 1,595 (2.65%, 95%CI 2.52–2.78%) patients. 1,129 patients (1.87%, 95%CI 1.77–1.98%) presented with a generator pocket infection leading to 2501 (3.20%, 95%CI 3.08–3.32%) related procedures. A lead-related endocarditis within three months from the index procedure was diagnosed in 466 (0.77%, 95%CI 0.71–0.85%) patients leading to 922 (1.18%, 95%CI 1.11–1.26%) related procedures. Overall, the likeliness to undergo a procedure related to CIED infection was higher in male patients, irrespective of generator pocket infection (69.4%, 95%CI 66.7–72.1%) or lead-related endocarditis (70.2%, 95%CI 65.9–74.2%). Patients presenting with CIED infection (median age 69.5 years) and lead related endocarditis (median age 67.7 years) were younger than patients undergoing any CIED procedure (median age 73.5 years) in that year. Despite the younger age upon occurrence of CIED infection, the mortality in case of major CIED pocket infection was greatly increased (8.4%), and after lead related endocarditis even more (15.24%). (Table 2)

**Table 2:** The dispersion of CIED infection and lead-related endocarditis within the cohort.

Major infection	Patients	Cases	Procedures (per case)	Male gender (%)	Median age	Deceased (%)
CIED-I	1,129	1,187	2,501 (2.11)	784 (69.4)	69.5	95 (8.4)
L-IE	466	477	922 (1.93)	327 (70.2)	67.7	71 (15.24)

CIED-I denotes CIED infection; L-IE denotes lead related endocarditis.

Hospitalization for CIED infection occurred in 784 of 34,523 male patients (2.27%, 95%CI 2.12 to 2.43%) and in 345 of 25,773 female patients (1.34%, 95%CI 1.21–1.49%).

The *gender* difference in the incidence of a localized CIED infection was 0.93% (95%CI 0.72–1.14%).

Hospitalization for lead related endocarditis occurred in 327 of 34,523 male patients (0.95%, 95%CI 0.85–1.05%) and in 139 of 25,773 female patients (0.54%, 95%CI 0.46–0.64%).

The *gender* difference in the incidence of a lead-related endocarditis was 0.41% (95%CI 0.27–0.54%).

The relative risk for major CIED infection in males was 1.7 (95%CI 1.5 to 1.9) that of females. Male gender displays a statistically significant risk factor for CIED infection.

**Fig. 2:**
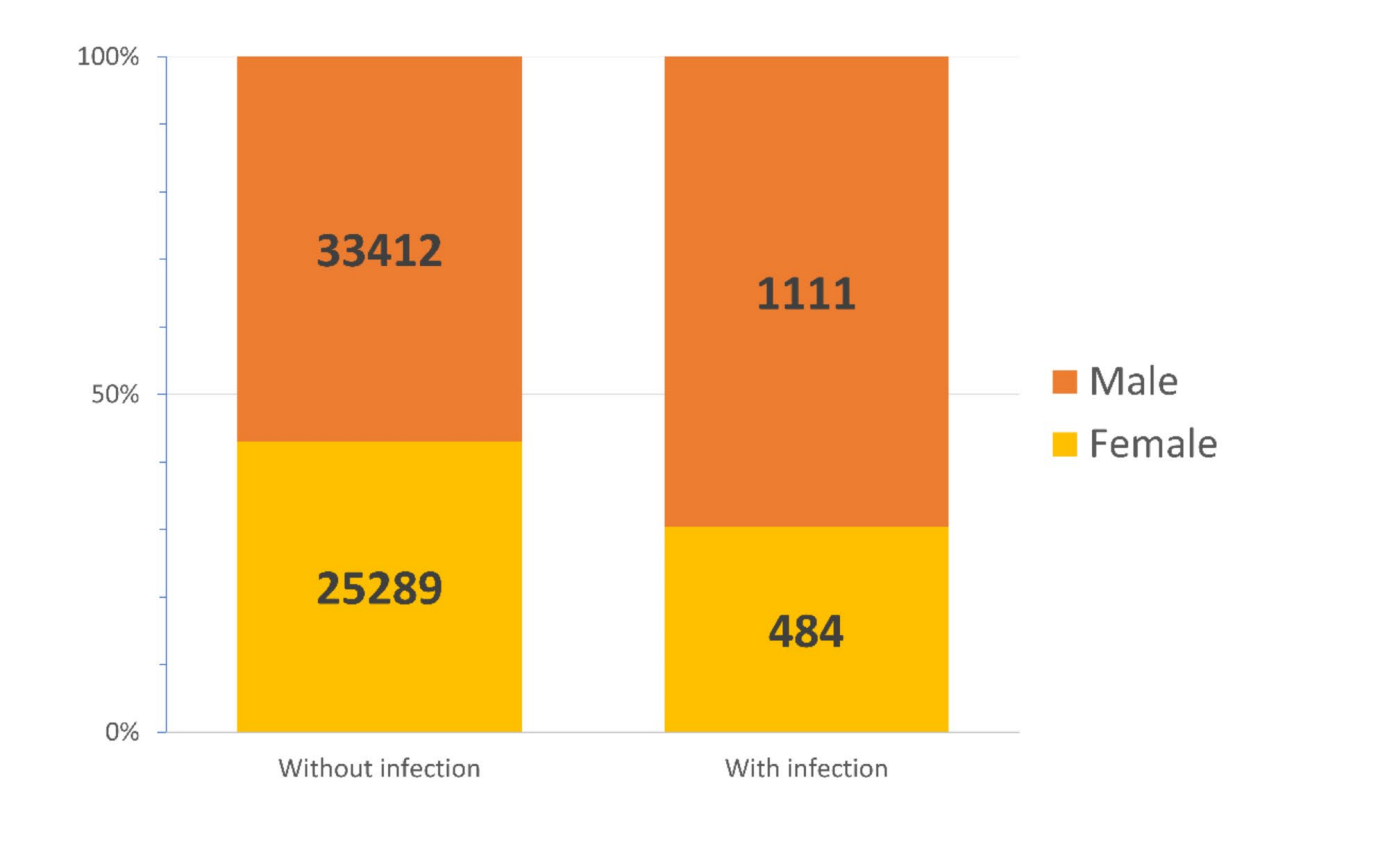
Gender distribution of major CIED infection.

Overall, the distribution of major CIED infections among all beneficiaries stratified by gender is reported in Fig. 2.

## Discussion

In a comprehensive analysis of a large German health insurance database, we identified 1,595 acute CIED infections (2.64%) among 60,296 patients undergoing CIED procedures in 2015, with 71% of these related to the generator pocket and 29% to lead-related endocarditis within 3 months of hospitalization.

Acute infections typically occur within the first few weeks to months following device implantation or revision surgery^7,8,22^. They often manifest as infections localized to the generator pocket with overt signs of infection such as erythema, warmth, tenderness, and purulent discharge at the surgical site, due to contamination during device implantation or pocket revision procedures. These infections may progress to involve the surrounding tissues or lead to systemic infection if left untreated^1,23^.

Lead related endocarditis refers specifically to infections involving the transvenous proportions of the leads of CIEDs, which can occur either in isolation or as part of a more extensive device infection. These infections are typically associated with bacteremia and may result from lead manipulation, erosion, or colonization^23,24^.

Our results indicate a higher prevalence of acute infection than previously reported, suggesting potential underdiagnosis in the wider community.

Contemporary literature highlights the substantial mortality rates associated with major CIED infections^2,25^. Mortality rates vary depending on factors such as the type and severity of infection, patient demographics, comorbidities, and timely intervention^12^. Studies have reported mortality rates ranging from 5 to 20% for major CIED infections, with higher rates observed in cases of endocarditis or systemic complications^11,26–28^.

In our study mortality rates were increased to 8.4% after pocket infection and 15.24% after endocarditis.

Comorbidities such as heart failure, diabetes, chronic kidney disease, chronic obstructive pulmonary disease, neoplasia or immunosuppression, can further increase the anticipated risk for CIED infection^10,29^.

Although our study did not investigate underlying comorbidities, we observed a significantly higher incidence of major CIED infections among male patients at the time of hospitalization. Additionally, patients who developed a major CIED infection tended to be younger.

Younger age and male gender are reported risk factors for CIED infection in different studies^10,25,30–32^. Various factors such as higher activity levels potentially leading to increased risk of trauma or injury, differences in immune response, and possibly hormonal influences are discussed. Additionally, anatomical differences and lifestyle factors may contribute to the higher susceptibility to infection in males. However, the exact reasons for these associations are not fully understood and further research is needed to elucidate the underlying mechanisms.

Although guidelines unequivocally establish the complete removal of all hardware as the gold standard, compliance with these recommended practices can vary significantly among healthcare providers and institutions^9,23,33^. Barriers to guideline adherence may include resource limitations, lack of awareness (patients^34^ and medical professionals), variability in clinical presentation, and individual clinician judgment^35^. A recently published study aimed to assess outcomes of Medicare patients with CIED infections. Only a small portion underwent device extraction, which was linked to lower mortality^24^.

In our study the median number of procedures per patient, including those for CIED infection and lead-related endocarditis, was 1.28 (1.25 per case reimbursed). Patients with pocket infection and lead-related endocarditis required more additional procedures, indicating a greater burden on healthcare resources and increased costs. These results suggest that immediate extraction of all hardware may not be consistently practiced according to current guidelines, potentially impacting patient outcomes and healthcare utilization.

CIED infections are a significant concern due to their associated morbidity and mortality. These infections can broadly be categorized into acute and late infections as well as lead related or generator pocket related, each with distinct clinical characteristics and management strategies^1,7,8,22,27^.

Late infections typically occur several months to years after device implantation and may be associated with indolent and nonspecific symptoms such as fatigue, malaise, or low-grade fever^22^. Late infections are often related to hematogenous seeding of the transvenous proportions of the device from a distant focus of infection or erosion of adjacent structures^36^. For involvement of the generator pocket contamination during the initial procedure with subsequent colonization and thus far unknown changes (i.e., disruption of an indwelling biofilm) leading to overt clinical infection are discussed^1^. Unless any hardware-protrusion through the skin leads to definite CIED infection diagnosis, identifying CIED involvement can be challenging due to subtle clinical presentations, and imaging modalities such as echocardiography and nuclear imaging may be necessary to confirm the diagnosis^9^.

Contemporary literature emphasizes the importance of a multidisciplinary approach involving infectious disease specialists, cardiologists, and cardiac surgeons in the diagnosis and management of CIED infections^1,23,24^. Early recognition, appropriate antimicrobial therapy, and timely device removal or extraction remain the cornerstone of successful treatment outcomes^1^. Additionally, advancements in imaging modalities and infection prevention strategies continue to refine our understanding and management of these complex infections.

## Limitations

Our investigation relies on de-identified billing data from the German health claims database, primarily designed for reimbursement purposes rather than epidemiological research. Data accuracy hinges on coding practices of healthcare facilities and may not encompass outpatient treatment. Thus, we acknowledge a potentially underestimated rate of CIED infections. The coding ambiguity of T82.7, encompassing various hardware infections, introduces complexity, as some patients with vascular grafts and CIEDs may be coded for vascular graft infections. Despite this, vascular prosthesis infections lead to bloodstream infections or hematogenous dissemination of pathogens, impacting transvenous proportions of the CIED.

Additionally, our findings might encompass minor CIED infections treated on an inpatient basis, such as superficial inflammation or wound dehiscence, but only if requiring local revision. A subset of patients may be readmitted solely for intravenous antibiotic therapy, potentially coded as CIED infection if applicable.

Our analyses were conducted on a fiscal year basis, acknowledging that patients undergoing index procedures late in 2015 may manifest CIED infections in subsequent years, potentially resulting in an underestimation of infection rates. Conversely, infections arising early in 2015 are likely attributable to primary placements or revisions performed in late 2014, thereby mitigating this effect. Thus, it is essential to view our study as a temporal snapshot of the situation.

We did not extend the observation period for this study, because this scenario holds the potential for multiple infection occurrences in one individual, such as coding for lead-related endocarditis, localized infection, minor infection, or infection of a vascular graft.

Understanding and stratifying the risk factors for infections associated with procedures, devices, and host-related elements reveals a fundamental challenge: as we attempt to dissect these influences further, the complexity of interactions begins to obscure clear associations. For instance, a skilled interventionalist may perform a revision with no resulting infection, while a less experienced clinician performing the same procedure might encounter complications, suggesting that the interplay of variables such as technique, procedural nuances, and patient-specific factors becomes diluted when analyzed at deeper levels of granularity. This dilution arises because the intricate web of influences - ranging from host immunity and device design to procedural conditions - cannot always be isolated effectively within a dataset. Consequently, while there is value in differentiating between factors like new implants versus revisions, the limitations of the current dataset prevent such granularity – a major drawback or limitation to our study. Instead, we emphasized the need to present an overview of infection rates to confront the overly optimistic everyday assumptions about their frequency. This highlights a trade-off between achieving detailed stratification and preserving the robustness of meaningful statistical inferences.

## Conclusions

In summary, our data clearly shows acute CIED infection has a greater prevalence than previously reported. Simultaneously, it may be conceivable that the adherence to guidelines for treating CIED infections might not meet the standard required to offer our patients the best chance of recovery. Male gender seems to be a relevant risk factor for development of major CIED infection. Additionally, major CIED infection occurred in younger individuals.

## Electronic supplementary material

Below is the link to the electronic supplementary material.

Supplementary Material 1

Supplementary Material 2

## Data Availability

The data for this study cannot be included in the manuscript, supplemental files, or a public repository due to German data protection laws (Bundesdatenschutzgesetz). They are securely stored at the “Wissenschaftliches Institut der AOK” for result replication. Access to statutory health insurance data for research is governed by German Social Law (SGB V § 287) and requires a formal proposal to the relevant data protection agency. External access to the study data is contingent upon the research project’s cooperation contract and AOK’s written approval. For access assistance, please contact andreas.kloess@wido.bv.aok.de.
