# Supplementary material for: Prevalence of cardiac implantable electronic device infections in Germany in 2015
Source: Sci Rep. 2024 Dec 16;14:30513. doi: 10.1038/s41598-024-82622-1 (PMC11649771; doi:10.1038/s41598-024-82622-1)
Supplement: Supplementary file 1 — Supplementary Material 1 [file 41598_2024_82622_MOESM1_ESM.docx]

**Table S1:** ICD-10 Codes (Version: German modification) and OPS Codes used

|  | Legend | | |
| --- | --- | --- | --- |
| CIED procedure codes | | | |
|  | 5-377 to 577.y | | Implantation PPM, ICD or event recorder |
|  | **OR** | |  |
|  | 5-378 to 5-378.d9 | | Extraction, exchange, correction, up- or downgrade of PPM or ICD |
|  | **OR** | | |
|  | 5-934.0 to 5-934.2 | | Use of MRI compatible PPM, ICD or event recorder |
| ICD-10 infection codes (German modification) | | | |
|  | (I33.0 to I33.9  **OR**  I38  **OR**  I39.0 to I39.8) | endocarditis  endocarditis not classified  endocarditis, valvular deterioration with underlying disease elsewhere | |
|  | T82.7 | Infection and inflammatory reaction caused by other devices, implants or grafts in the heart and blood vessels | |
