# Supplementary material for: Prevalence of cardiac implantable electronic device infections in Germany in 2015
Source: Sci Rep. 2024 Dec 16;14:30513. doi: 10.1038/s41598-024-82622-1 (PMC11649771; doi:10.1038/s41598-024-82622-1)
Supplement: Supplementary file 2 — Supplementary Material 2 [file 41598_2024_82622_MOESM2_ESM.docx]

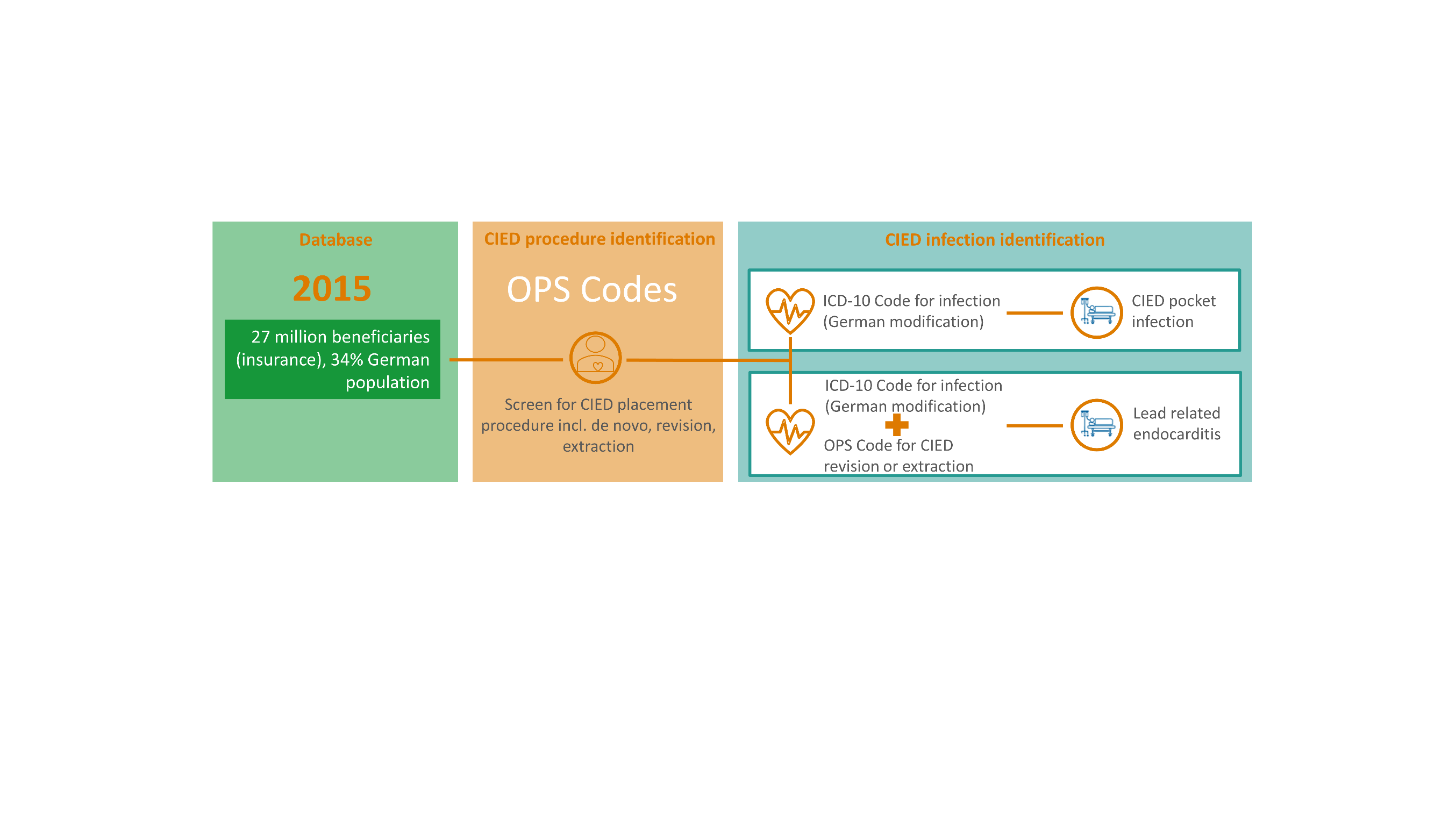


**Figure S1:** Algorithm for identification of CIED procedures and CIED related infections and lead-related endocarditis in our cohort
